# NF-κB-Dependent Production of ROS and Restriction of HSV-1 Infection in U937 Monocytic Cells

**DOI:** 10.3390/v11050428

**Published:** 2019-05-10

**Authors:** Francesca Marino-Merlo, Emanuela Papaianni, Caterina Frezza, Silvana Pedatella, Mauro De Nisco, Beatrice Macchi, Sandro Grelli, Antonio Mastino

**Affiliations:** 1 IRCCS Centro Neurolesi Bonino-Pulejo, 98123 Messina, Italy; fmarino@unime.it; 2 Department of Chemical, Biological, Pharmaceutical, and Environmental Sciences, University of Messina, 98166 Messina, Italy; epapaianni@unime.it (E.P.); catafrezza@hotmail.it (C.F.); 3 Department of Chemical Sciences, University of Naples “Federico II”, 80126 Naples, Italy; pedatell@unina.it; 4 Department of Science, University of Basilicata, 85100 Potenza, Italy; mauro.denisco@libero.it; 5 Department of Chemical Science and Technologies, University of Rome “Tor Vergata”, 00133 Rome, Italy; macchi@med.uniroma2.it; 6 Department of Experimental Medicine, University of Rome “Tor Vergata”, 00133 Rome, Italy; grelli@med.uniroma2.it; 7 The Institute of Translational Pharmacology, CNR, 00133 Rome, Italy

**Keywords:** HSV-1, oxidative stress, NF-κB, innate response

## Abstract

Herpes simplex virus 1 (HSV-1) can infect a wide range of cell types, including cells of the adaptive and innate immunity but, normally, it completes a fully-permissive replication cycle only in epithelial or neural cells. Complex mechanisms controlling this delicate balance in immune cells and consequent restriction of HSV-1 infection in these cells have not been completely elucidated. We have recently demonstrated that the transcription factor nuclear factor kappa B (NF-κB) can act as a main permissiveness regulator of HSV-1 infection in monocytic cells, however, mediators involved in this regulation have not been identified. To better define mechanisms involved in this phenomenon and, particularly, the possible involvement of ROS, wild type U937 cells or U937 cells stably transfected with a dominant-negative (DN) IκB-mutant and selenium-containing compounds, as anti-oxidants, were utilized. The main results can be summarized as follows. HSV-1 infection induces an immediate ROS production in U937 monocytic cells that can efficiently activate NF-κB but not in DN-IκB-mutant cells. Treatment with selenium-containing antioxidants efficiently inhibited HSV-1-induced ROS generation while producing increased levels of HSV-1 replication and a reduction of HSV-1-induced NF-κB activation in U937 monocytic cells. Our results suggest a scenario in which an efficient NF-κB-dependent ROS production in response to infection could contribute in limiting HSV-1 replication in monocytes/macrophages, thus avoiding possible irreparable damage to the innate immune system of the host during HSV-1 infection.

## 1. Introduction

Herpes simplex virus 1 (HSV-1) has coexisted with modern humans since the beginning of their appearance on Earth. In fact, HSV-1 co-diverged from its ancestor virus concurrently with modern humans from primates [1]. Such a long-term co-evolution has resulted in a balanced and complex mechanism of persistent infection, characterized by latency and reactivation, which is suitable for, under normal conditions, the survival of both the human host and the virus [2]. Persistent infection by HSV-1 and its closely related herpes simplex virus 2 (HSV-2) in healthy individuals is ensured by a number of regulated interactions among elements of the host response and of the evasion strategies of the virus. Nevertheless, a dysregulation of these interactions could transform a mild and often asymptomatic viral infection into a dramatic and life-threatening disease such as encephalitis, as also highlighted by murine experimental models [3,4]. Interestingly, studies by different authors have highlighted a number of complex interactions among HSV-1 or HSV-2 herpes simplex viruses (HSV) and components of the innate antiviral response, such as regulated cell death and the transcription factor nuclear factor kappa B (NF-κB) [5,6,7,8,9,10,11,12,13,14,15]. One peculiarity of the regulated virus/cell interactions occurring during infections by HSV is that they greatly differ according to the type of infected cell. The three main, different types of relationships that HSV establish with infected cells can be principally distinguished as lytic infection, typical of epithelial cells, latent infection, typical of neurons, and low-productive or non-productive infection, which is typical of mononuclear cells of the adaptive or innate immunity. The low or non-productive infection [16,17] is the less studied and characterized form of HSV infection and can evolve differently according to the specific cell type and its differentiation stage. Particularly, HSV infection of monocytes/macrophages is generally characterized by the fact that HSV can efficiently enter target cells and initiate the replication cycle. Then, the replication cycle is immediately interrupted (non-productive or abortive infection) or, at least, partially restricted, originating a low-productive, but still well-detectable, semi-permissive replication cycle [18]. Recently, we demonstrated that activation of NF-κB during HSV-1 infection, differently from what occurs in fully permissive cells where it has been hijacked to a pro-virus factor, plays a central role in restriction of infection in monocytic cells, preserving these cells from both viral replication and apoptosis [19]. In fact, viral production, as assayed by different techniques, as well as levels of apoptotic regulated cell death, that proved to be quite low in spite of high-dose virus challenge in wild type U937 cells, were dramatically increased in U937 cells in which NF-κB activation was stably inhibited by a dominant-negative IκBα, with respect to control U937 cells [19]. Intriguingly, however, none of the investigated NF-κB-dependent genes, most commonly associated with the antiviral response, seemed to be involved in restriction of HSV-1 infection in monocytic cells and underlying mechanisms have not yet been completely elucidated [19].

Emerging data indicate that production of reactive oxygen species (ROS) plays beneficial effects in several biological functions, including innate immunity and antiviral responses. It has long been known that ROS act as a microbicidal compound produced by NADPH oxidases (NOXs) in phagosomes of phagocytic cell types [20]. Moreover, ROS have been shown to regulate a number of processes involved in the innate response, such as expression of innate-response-related genes, signal transduction, inflammasome activation, autophagy and programmed necrosis [21,22]. However, the role of ROS production during viral infections is still controversial. In fact, a lot of data have been accumulated over the past years showing that treatment with anti-oxidants could be beneficial against infections caused by different viruses. Actually, results reported in different studies indicate that in the case of HSV-1 infection, ROS production could exert an antiviral effect [23,24,25,26]. Interestingly, overexpression of IκBα has been demonstrated to inhibit inducible ROS production, showing also that this presumable component of the innate response could be under the control of NF-κB. However, the contribution of a possible crosstalk between NF-κB signalling and ROS production in innate immune response to HSV-1 infection in monocytic cells remains largely elusive. 

The present study was aimed to further elucidate the relationships among ROS production, NF-κB activation and viral replication in monocytic cells infected by HSV-1. For this purpose, we used U937 monocytic cells in which NF-κB activation was functional or, in parallel, ablated by expression of a dominant-negative IκBα. Moreover, to inhibit ROS production, we used selenium-containing compounds whose anti-oxidant and biological properties have been previously described [27].

## 2. Materials and Methods

### 2.1. Cells 

Human monocytic wild type U937 cells, originally obtained from the Istituto Zooprofilattico, Brescia, Italy, and U937 cells in which NF-κB activation was abrogated by expression of a dominant negative form of murine IκBα (DN-IκB) to produce stable transfectants, were grown and cultured as previously described [28], and checked by EMSA for inhibition of NF-κB activation. HEp-2 and THP-1 cells, originally obtained from American Type Culture Collection (ATCC), were cultured in RPMI 1640 (Lonza, Basel, Switzerland) supplemented with 10% fetal bovine serum (FBS, Euroclone, Milan, Italy). All media were supplemented with 100 units/mL penicillin, 100 mg/mL streptomycin and 2 mM L-glutamine (Lonza). Medium for THP-1 cells was also supplemented with 1 mM sodium pyruvate, 10 mM Hepes and 4.5 g l-1 glucose (final concentration). Cell lines were cultured at 37 °C in a 5% CO_2_ incubator. 

### 2.2. Antibodies and Reagents 

Mouse monoclonal antibodies against HSV-1 gD DL6 (sc-21719) and HSV-1 ICP0 (sc-53070) were obtained from Santa Cruz Biotechnology (Santa Cruz, CA, USA); mouse anti-human β-actin (mAbcam 8226) from Abcam (Cambridge, UK). The secondary fluorescein isothiocyanate (FITC)-conjugated and horseradish peroxidase (HRP)-conjugated anti mouse IgG antibodies were obtained from EMD Millipore (Temecula, CA, USA).

Se-(Methyl)selenocysteine hydrochloride (SeMC, M6680), the oxidant-sensitive dye 2′,7′-dichlorofluorescin diacetate (DCFH-DA, D6883), H_2_O_2_ (H1009) and cycloheximide (CHX, 01810) were purchased from Sigma-Aldrich (St. Louis, MO, USA). A second selenium-based compound used in this study and identified as Seleno-Diamino Acid 4 (SeDA4), is a synthetic molecule obtained and characterized for its anti-oxidant and biological properties as previously described [27]. 

### 2.3. Virus, Infections and Treatments

HSV-1 virus strain F, originally obtained from ATCC, was propagated, quantified and stored as described below. Briefly, to obtain virus stocks, sub-confluent monolayers of Vero cells were inoculated with HSV-1 F at a multiplicity of infection (MOI) of 0.01 for 2 h at 37 °C in DMEM supplemented with 1% FBS. Virus inoculum was then removed, fresh DMEM supplemented with 5% FBS was added, and the cells were incubated at 37 °C in 5% CO_2_. When a cytopathic effect of 100% was ascertained, additional FBS was added (20% final concentration) and infected cultures were frozen and thawed three times. Cell debris was then removed by centrifugation at 2500 RPM for 30 min at 4 °C. Virus titers in clarified supernatants were determined by plaque assay. Virus stocks were kept at −80 °C until use. Monocytic cells and adherent HEp-2 cells were split 24 h before infection and then either mock infected or exposed to indicated multiplicity of infection (MOI) of HSV-1 for 1 h at 37 °C. After the adsorption period, the virus inoculum was replaced with RPMI 1640 containing 1% FBS and cultures were further incubated at 37 °C for 8, 16 or 20 h post infection (p.i.). For short-time kinetics experiments, some samples were collected during the adsorption period. In these cases, the time from initial contact with virus has been reported as time of exposure to HSV-1.

For modulation of ROS production, cells were pre-incubated with 20 µM of selenium-based compounds for 48 h, 5 µM of H_2_O_2_ for 0.5 h, or with vehicle alone as a control for 48 h or 0.5 h. All samples were washed three times in complete fresh medium and then infected with HSV-1, as described above. In some experiments, the combination of ROS-modulating agents and oxidative stress induction was used. In this case, vehicle- and SeMC- or SeDA4-treated cells were incubated for a further 0.5 h with H_2_O_2_ 5 µM or vehicle prior to infection. The time of treatments and the concentrations used were chosen based on preliminary experiments performed by trypan blue exclusion to select the optimal experimental conditions, i.e., undoubtedly non-cytotoxic concentration ranges of SeMC, SeDA4 and H_2_O_2_ towards U937 and HEp-2 cells.

For inhibition of *de novo* protein synthesis, U937 cells were pretreated with 1% FBS phenol-red-free RPMI containing CHX (1 μg/mL), or equal volumes of DMSO as a control, for 1 h at 37 °C. Twenty minutes before the end of CHX pretreatment, DCFH-DA was added to a final concentration of 10 µM. After washing, cells were infected with HSV-1 at a MOI 50 for 30 min before microscope analysis. Concentration of CHX to utilize was selected based on preliminary dose-response experiments that excluded toxicity and proved efficacy in inhibiting de novo protein synthesis in HSV-1-infected U937 cells for 1 μg/mL CHX at the chosen experimental conditions.

### 2.4. ROS Detection 

Intracellular ROS level was determined using the 2′,7′-dichlorofluorescin diacetate (DCFH-DA), which is a cell permeable and nonfluorescent agent that can be deacetylated by intracellular esterases to non-fluorescent DCFH. In the presence of ROS, DCFH is converted intracellularly to the oxidized fluorescent form, DCF. Cells were shifted to phenol-red-free RPMI with reduced serum (1%) and preloaded with DCFH-DA 10 µM at 37 °C for 30 min before HSV-1 infection. At the designated time point, cells were washed with PBS and immediately analyzed by Leica DMR fluorescence microscopy (Leitz, Wetzlar, Germany) or by the Observer Z1 fluorescence microscope (Zeiss, Jena, Germany), where indicated. For kinetics of virus exposure from 0.5 h to 2 h, cells were incubated with the probe at the same time, washed and HSV-1-infected or mock-infected with different starting-points to analyze all samples and relative fluorescent signals simultaneously. For each experiment, as a positive control, a preload DCFH-DA sample treated with H_2_O_2_ 10 µM for 0.5 h was added. In preliminary and parallel experiments, cells were also loaded with the probe at the end of the infection period and imaged immediately after. No differences in the detectability of the pre- or post-loaded probe for incubation periods until 4 h were noted but reduced background fluorescence in images taken from preloaded samples was found.

For quantitative evaluation of ROS positive cells, digital images, collected with brightfield or FITC filter using 40× or 63× objectives, were analysed by ImageJ algorithm software (NIH, Bethesda, MD, USA). For each frame, background fluorescence was eliminated and an arbitrary fixed threshold was set. Resulting green fluorescent positive cells were counted and percentage of DCF fluorescent cells relative to the total number of cells per frame, obtained in a corresponding acquired brightfield, was calculated. Data obtained from at least six randomly selected frames from at least two separate experiments were evaluated per condition. A minimum of 100 cells per frame were analysed. Some representative images were also taken by a 20× objective. 

### 2.5. Immunofluorescence Microscopy Analysis

For gD detection by immunofluorescence microscopy analysis, experimental cultures were collected 20 h post infection, and cells were fixed and stained with mouse anti-gD HSV-1 specific antibody and with Hoechst 33342 as previously described [19]. Adherently growing epithelial HEp-2 cells were cultivated, pre-treated, infected with HSV-1 and processed directly on multi-well slides. Images were collected using Leica DMR fluorescence microscopy with a 40× objective in a green filter for FITC-labeled antibody and in a blue filter for Hoechst stained nuclei. For quantification, green fluorescent gD-positive cells and total blue fluorescent nucleated cells were counted in each captured field. Amounts of gD positivity were represented as a percentage of positive cells calculated in ten randomly selected fields from three independent experiments.

### 2.6. Virus Titer (Plaque Formation) Assay

Virus titer was determined from supernatants of each infected culture by standard plaque assay on Vero cells as previously described [29]. Results were expressed as the percent yield of HSV-1 with respect to the corresponding control sample (% HSV-1 yield = PFU from infected pre-treated sample/PFU from infected vehicle-pre-treated sample × 100).

### 2.7. Western Blot Analysis

Expression of the immediate-early protein (ICP0) and of a late protein (gD) of HSV-1 in U937 infected and mock-infected samples was evaluated, respectively, at 8 h and 16 h p.i. by western blotting. Cells were lysed in NP-40 lysis buffer containing protease inhibitors (Roche Applied Science, Penzerberg, Germany), and the lysates were electrophoresed through denaturing 10% SDS-polyacrylamide gel. Proteins were transferred onto a nitrocellulose membrane (Bio-Rad, Hercules, CA, USA), followed by block of non-specific binding with 5% non-fat milk in Tris-buffered saline (TBS) containing Tween 0.1%. Membrane was then incubated with primary antibodies, anti-gD, anti-ICP0 (Santa Cruz) and anti β-actin (Abcam), and then with HRP-conjugated secondary antibodies, followed by incubation with Pico-Sensitivity ECL (Thermo Scientific, Waltham, MA, USA). The ImageJ software was utilized to quantify densitometric values of blots [19].

### 2.8. NF-kB Binding Assay

For detecting NF-kB activity, nuclear extracts from U937 cells in the different experimental conditions were assayed by non-radioactive electro mobility shift assay (EMSA). Binding reactions were performed by incubating 10 μg of nuclear protein extracts and 5 pmol of biotin-labeled kB DNA probe for 20 min at room temperature [30,31]. Complexes were resolved by non-denaturing 5% polyacrylamide gel electrophoresis and then transferred to a positive charge nylon membrane (Bio-Rad). Signals from the biotin-labeled probe were detected using LightShift™ Chemiluminescent EMSA Kit (Pierce, Thermo Fisher Scientific). Densitometric evaluation of scanned films from two separate experiments by Image J software was performed to quantify NFκB DNA binding activity. Data were represented as relative density calculated by the ratio between values obtained for all samples and values of mock vehicle-pre-treated samples.

### 2.9. Isolation of Total RNA and Real-Time qPCR

The total RNA extraction and real time RT-qPCR analysis procedures were described previously [19]. Forward (F) and reverse (R) sequences of the primers selected for Nox family genes by using Primer3 software, are reported in Table 1.

### 2.10. Statistical Analysis

The data were expressed as mean ± standard deviation (SD). For each series of experiments, two to four independent replicates were performed. Statistical significance was determined by Dunnett’s multiple comparison test, using Prism 5 (GraphPad Software, San Diego, CA, USA). A *p*-value < 0.05 was considered to indicate statistical significance.

## 3. Results

### 3.1. HSV-1 Infection Induces an Early ROS Production in Monocytic Cells

**Figure 1:**
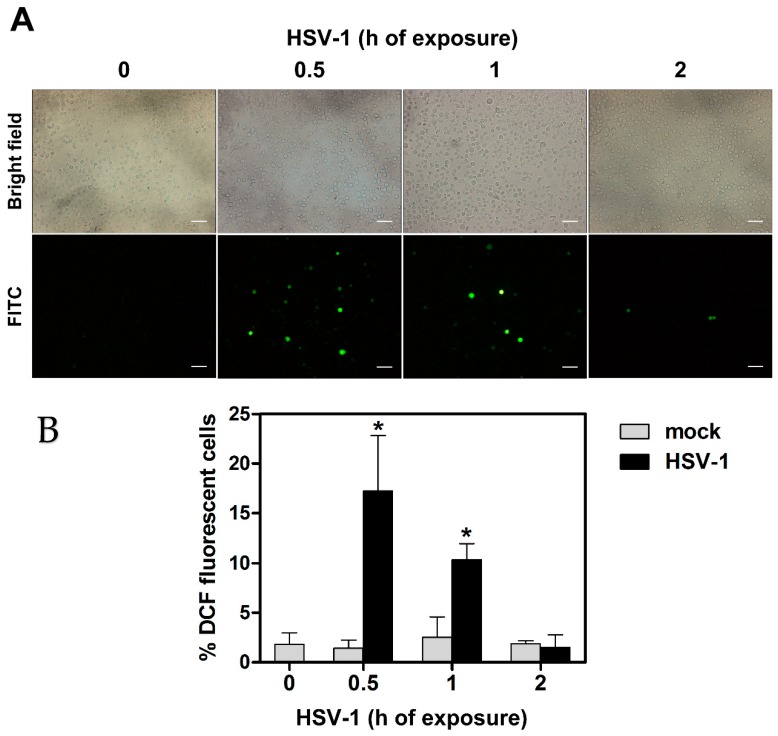
ROS production in U937 cells exposed to HSV-1. Cells were preloaded with DCFH-DA 10 µM before HSV-1 infection (MOI = 50 PFU/cell). At indicated times post virus exposure, ROS production was monitored by fluorescence microscopy. (**A**) Brightfield (total cells) and FITC channel (ROS positive cells) from representative images. 20× objective, scale bar = 50 μm. (**B**) % of DCF fluorescence positive cells (mean + S.D). Data were obtained from 6 frames randomly selected from three separate experiments (2 frames each). Digital images were captured by 40× or 63× objectives. * *p* < 0.001. vs control (h 0).

We have preliminarily checked whether HSV-1 infection resulted in production of ROS in our experimental model of infection of monocytic cells, consisting of wild type U937 cells. To this purpose U937 cells were preloaded with a non-fluorescent agent that could be converted intracellularly to a fluorescent probe when oxidized by ROS, and then infected by HSV-1 at a MOI of 50 PFU/cell. At different times, p.i. cells were collected, washed, distributed on multi-well slides and ROS production was assessed by evaluating the presence of cells harboring the fluorescent probe by fluorescence microscopy analysis. Results of one representative analysis is reported in Figure 1a. Appearance of well-detectable fluorescent cells was easily appreciable as early as 30 min p.i. in the HSV-1 infected sample. Fluorescent cells were still well detectable at 1 h p.i., but not at 2 h p.i. Results can be quantitatively appreciated in Figure 1b, where histograms refer to the mean percentages of fluorescent cells + S.D., as calculated from three independent experiments, with two fields each. Preliminary experiments showed that infections with a MOI of 5, 10 or 20 PFU/cell induced lower levels of ROS-producing cells, which were not significant with respect to control cells at the two lower concentrations assayed. Results of these experiments clearly confirmed the capacity of HSV-1 to induce an immediate oxidative stress response, unequivocally disclosed by ROS production, after infection of monocytic cells. Moreover, these results indicated that the ROS response to infection was transient and limited to the very early phase of infection.

### 3.2. HSV-1-Induced ROS Production and Expression of Oxidant Genes Are Dependent on NF-kB Activation in Monocytic Cells

**Figure 2:**
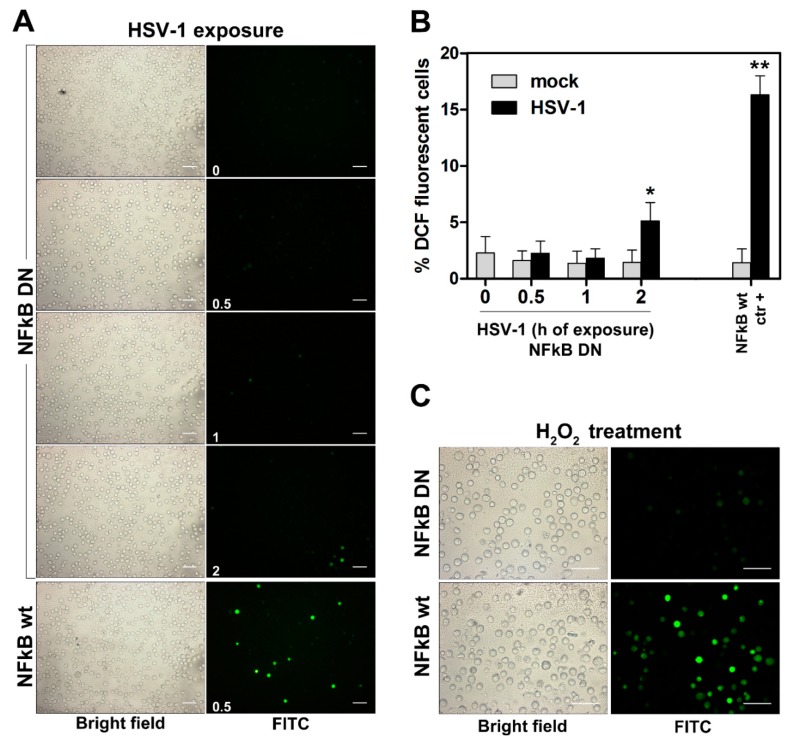
Effects of NF-kB ablation on ROS production in U937 cells. DCFH-DA preloaded U937-DN-IkB (NFkB DN) were exposed to HSV-1 (MOI = 50 PFU/cell) or vehicle alone (mock) from 0.5 to 2 h for ROS detection by fluorescence microscopy. In each experiment, DCFH-DA preloaded wt U937 cells (NFkB wt), exposed or mock exposed to HSV-1 for 0.5 h, were also included as positive controls. (**A**) Representative images by 20× objective. Times of HSV-1 exposure (h) are reported in the FITC frames (0 = mock infected U937-DN-IkB). Scale bar = 50 μm. (**B**) % of DCF fluorescence positive cells (mean + S.D) from 8 frames randomly selected from two independent experiments (4 frames each). * *p* < 0.01, ** *p* < 0.001 vs. control (h = 0). (**C**) DCFH-DA preloaded NFkB DN or wt U937 cells, were treated with 5 µM H_2_O_2_ for a further 30 min and then washed and analysed for ROS production by fluorescence microscopy. Objective 40×, scale bar = 50 μm.

Results from us and other authors established that NF-κB activation represents a very early event in the cellular response to HSV-1 infection. In addition, it is known that ROS production could be affected either positively or negatively by NF-κB activation, depending on implicated variables. Thus, we asked whether an interplay could exist among NF-κB activation and ROS production in response to HSV-1 infection in monocytic cells. We, then, firstly investigated the effects of inhibition of NF-κB activation on ROS production in U937 cells. To this purpose we took advantage by the availability in our laboratory of a U937-derived cell line in which NF-κB activation was ablated by stable transfection with a DN-IκB. Wild type U937 cells and DN-IκB U937 cells were then infected and ROS production was detected. Results, shown in Figure 2A,B revealed that, surprisingly, ablation of NF-κB activation completely blocked the ROS-response to HSV-1 infection in monocytic cells at early times tested, while a quite low percentage of cells in which ROS production could be detected was observed at 2 h p.i. We asked whether the NF-κB-dependency of the oxidative stress response in U937 cells was limited to HSV-1 as an inducing agent. To answer this question, we treated wild type and DN-IκB U937 cells with H_2_O_2_. Interestingly, also in this case the oxidative stress was dramatically suppressed in cells in which NF-κB activation was abrogated.

**Figure 3:**
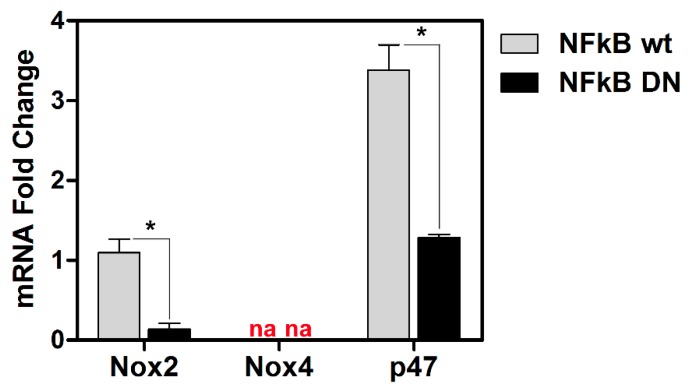
Effects of NF-kB ablation on basal expression of the indicated “oxidant” genes. mRNA levels of Nox2 and Nox4 isoforms and p47 Nox complex component, quantified by qPCR in wt or NFkB DN U937 cells. Data were normalized to 18S rRNA and results are expressed as relative fold change versus Nox2 mRNA levels in wt control cells. Mean values + S.D. from three independent experiments performed in duplicates are depicted. * *p* < 0.001. na: no amplified cDNA detected.

ROS production is regulated by specific oxidant genes. We then examined basal levels of transcriptional activity for three representative genes notoriously involved in the oxidative response, such as NOX2, NOX4 and p47 in wild type and DN-IκB U937 cells. Figure 3 shows that basal mRNA levels for NOX2 and p47 were highly reduced in DN-IκB cells with respect to control cells. Basal levels of NOX4 were not detectable both in control and DN-IκB U937 cells. Taken together, these results account for a clear NF-κB-dependency of the ROS response to HSV-1 infection in monocytic cells, thus revealing a strict interplay among the above mentioned factors.

### 3.3. HSV-1-Induced ROS Production in Monocytic Cells Is Not Cell-Line Specific and Does Not Depend on Protein Neo-Synthesis

**Figure 4:**
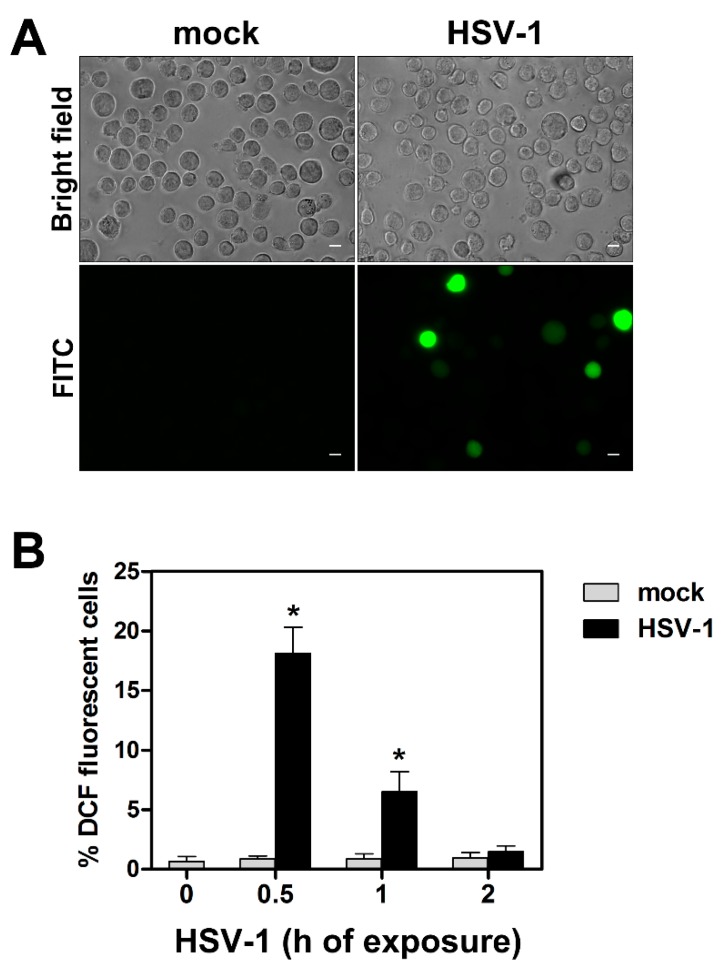
ROS production in THP-1 cells exposed to HSV-1. Cells were preloaded with DCFH-DA 10 µM, infected with HSV-1 and monitored for ROS production as described in Figure 1. (**A**) Brightfield (total cells) and FITC channel (ROS positive cells) from THP-1 cells infected or mock-infected with HSV-1, MOI 50, for 0.5 h. Digital images were captured by a 40× objective (Zeiss Axio Observer Z1 microscope). Scale bar = 10 μm. (**B**) % of DCF fluorescence positive cells (mean + S.D) obtained by fluorescence microscope analysis. Frames randomly captured by 20× objective from two separate experiments (2 frames each, total n = 4) were evaluated. * *p* < 0.001. vs control (h 0).

Having observed that HSV-1 infection caused an immediate ROS production in U937 monocytic cells, we asked whether: (i) this effect was restricted to the specific cell line utilized or could be extended to other monocytic cells, (ii) production of ROS was dependent on *de-novo* protein synthesis. To answer the first question, we repeated our experiments of ROS detection in human monocytic THP-1 cells. Also in this case, cells were preloaded for ROS detection by the fluorescent probe and then infected by HSV-1 at a MOI of 50 PFU/cell. At different times, p.i. cells were collected, washed, distributed on multi-well slides and ROS production was assessed as for U937 cells. Results of one representative analysis is reported in Figure 4a, showing the appearance of well-detectable fluorescent cells as early as at 30 min p.i. in the HSV-1 infected sample, as occurred for U937 cells. Results at all times tested can be quantitatively appreciated in Figure 4b, and recapitulate what was observed for U937 cells.

**Figure 5:**
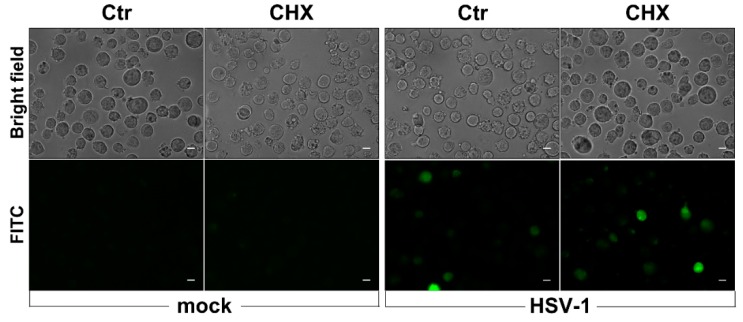
Effects of cycloheximide (CHX) treatment on early ROS induction by HSV-1. U937 cells, pretreated with CHX 1 µg/mL or with vehicle alone (Ctr) for 1 h, were loaded with DCFH-DA before exposure to HSV-1 (MOI = 50 PFU/cell) for further 30 min and successive fluorescence microscopy analysis. Representative images in bright-field and FITC channel are shown (40× objective, Zeiss Observer Z1 microscope). Scale bar Scale bar = 10 μm.

In addition, to verify whether prompt ROS production in response to HSV-1 infection required protein neosynthesis, U937 cells were infected in the presence of the protein synthesis inhibitor cycloheximide. Results shown in Figure 5 indicate that no change in ROS-induced fluorescent cells could be observed following CHX treatment in comparison with control samples, demonstrating that early ROS production in response to HSV-1 infection of monocytic cells is not dependent on protein neosynthesis. 

### 3.4. Selenium-Based Compounds Inhibit ROS Production in Monocytic Cells Infected by HSV-1

**Figure 6:**
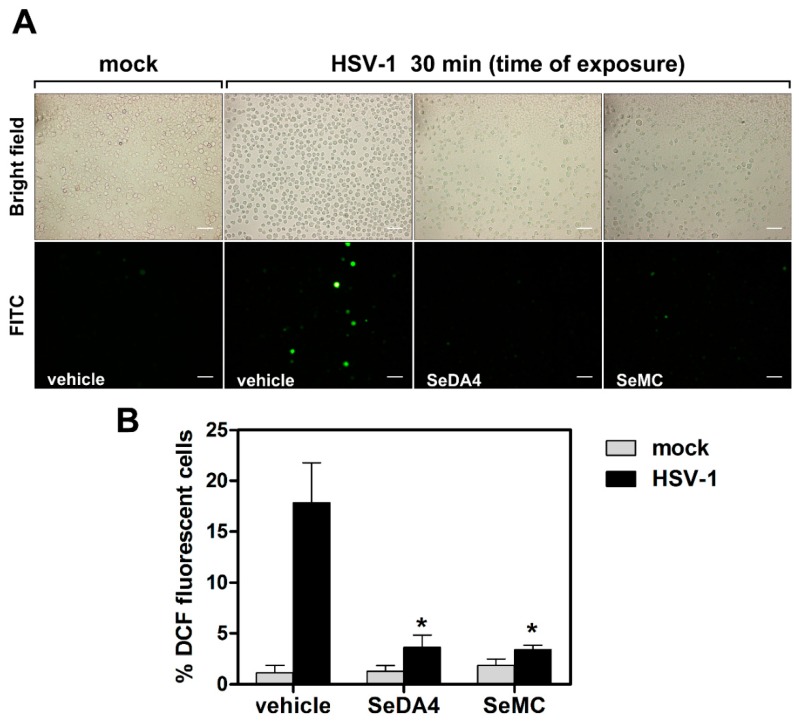
Effects of selenium-based antioxidants on early ROS induction by HSV-1. U937 cells were incubated with SeDA4 (20 µM), SeMC (20 µM) or vehicle alone for 48 h and then preloaded with DCFH-DA before exposure to HSV-1 (MOI = 50 PFU/cell) for a further 30 min and successive fluorescence microscopy analysis. (**A**) Representative images in bright-field (upper panels) and FITC (bottom panels) of cells subjected to different experimental conditions are displayed. Objective 20×, scale bar = 50 μm. (**B**) % of DCF fluorescence positive cells (mean + S.D) from 6 frames randomly selected from two independent experiments (3 frames each). * *p* < 0.001 vs. control HSV-1-infected cells (vehicle).

The following question was to ascertain whether the HSV-1-induced ROS production in monocytic cells could be affected by compounds endowed with anti-oxidant activities. To this purpose we utilized two members of a family of compounds characterized by the presence of selenium in their amino acidic aromatic rings. The biological properties of these compounds, whose anti-oxidant activities were already documented, have been previously described [27]. In particular, we utilized SeMC, as a reference commercial compound, and SeDA4, as a newly in-house synthesized compound. U937 cells were pretreated at non-toxic concentrations of SeMC and SeDA4 before loading with the revealing probe, infection with HSV-1 and successive detection of ROS. As shown in Figure 6, pretreatment with the selenium-based compounds greatly suppressed the ROS production induced by HSV-1 infection in U937 cells.

### 3.5. Inhibition of ROS Production Promotes Viral Replication in HSV-1 Infected Monocytic Cells in a NF-κB-Dependent Manner 

**Figure 7:**
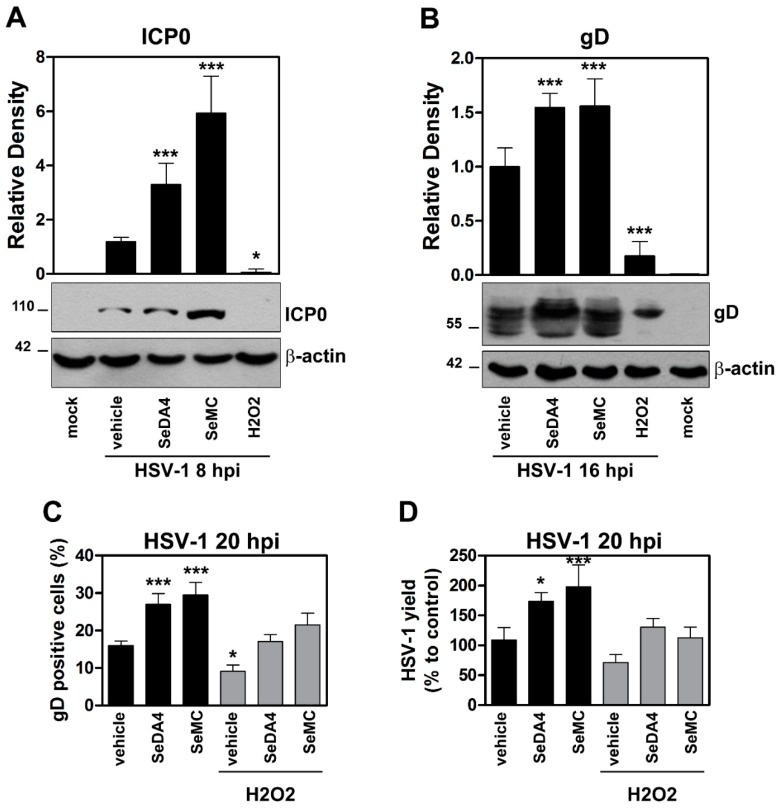
Effects of ROS-modulating agents on HSV-1 replication. U937 cells were pre-incubated with selenium-based antioxidants (SeDA4 or SeMC 20 µM) for 48 h and/or exposed to H_2_O_2_ for a further 30 min before infection with HSV-1 (MOI 50 PFU/cell). At the indicated times, cells (hpi) were collected and assayed for virus replication by different techniques. (**A**,**B**) Western blot analysis of ICPO (**A**) and gD (**B**) viral proteins from samples pre-incubated with antioxidants or complete media (vehicle) before exposure to H_2_O_2_ and infection. Mock-infected cultures were also included. Top panels: densitometric analysis normalized to β-actin expression and expressed as mean + S.D of data obtained from two evaluations in duplicates. Bottom panels: representative gels. (**C**) Immunofluorescence microscopy analysis of gD expressing cells from samples pre-incubated with antioxidants or complete medium (vehicle) before exposure to H_2_O_2_ and infection. Analysis by IFA is expressed as % positive cells (mean + S.D.) calculated from 20 different fields per conditions of four separate experiments (5 fields each). (**D**) Virus yield in supernatants determined by plaque assay in samples obtained from the same experiments of C. Data are expressed as percentage of HSV-1 yield with respect to infected, untreated cells (vehicle). * *p* < 0.05, ** *p* < 0.001, *** *p* < 0.0001 versus HSV-1-vehicle treated groups (only infected groups are represented).

**Figure 8:**
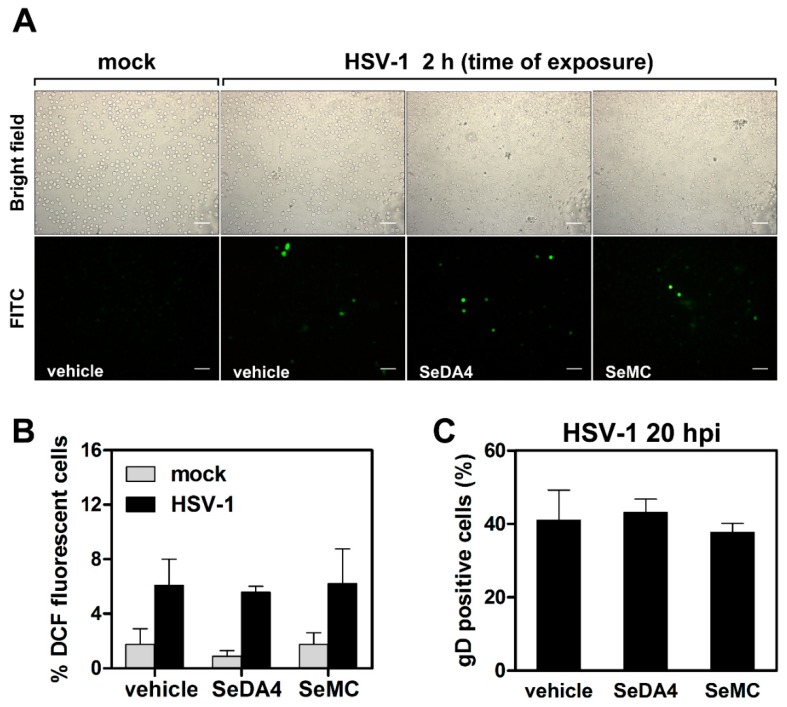
Effects of selenium-based antioxidants on monocytic cells with abrogated NF-KB activity and exposed to HSV-1. U937-DN-IkB were incubated with SeDA4 (20 µM), SeMC (20 µM) or vehicle alone for 48 h and then preloaded with DCFH-DA before infection with HSV-1 (MOI = 50 PFU/cell) and successive fluorescence microscopy analysis. **(A)** Representative images of cells collected at 2 h post infection. 20× objective. Scale bar = 50 μm. (**B**) Quantification of results shown in A by analyses from 8 frames randomly selected from two separate experiments (4 frames each). (**C**) Evaluation of gD-HSV-1 expressing cells by immunofluorescence microscopy analysis of cells collected at 20 h post infection. Each bar represents the mean value + S.D. of three independent experiments. No significant differences among infected cells either for ROS production (**B**) or for infectivity rate (**C**).

We then wanted to investigate whether the interplay among ROS production and NF-κB activation could affect HSV-1 infection in monocytic cells. To this purpose, we monitored the progression of HSV-1 replication in control and DN-IκB U937 cells, pretreated or not with the selenium-based antioxidants. Figure 7 reports results obtained in wild type, NF-κB activation-competent, U937 cells. The progression of HSV-1 replication was monitored by several techniques, including: western blot analysis of the immediate early ICP0 viral protein at 8 h p.i. (Figure 7A), western blot analysis of the late gD viral protein at 16 h p.i. (Figure 7B), detection of the gD protein by fluorescence microscopy at 20 h p.i. (Figure 5C), and detection of virus yield from infected cultures (Figure 7D). Results obtained using the four different methods coherently demonstrated that pre-treatment with the SeDA4 and SeMC unequivocally facilitated the progression of infection. This result revealed that ROS production during the early phases of HSV-1 infection exerted an inhibitory effect towards viral replication. Interestingly, this effect was well detectable even for the immediate early ICP0 protein synthesis, as early as at 8 h p.i. Moreover, when the oxidative stress was super-induced by addition of H_2_O_2_ 30 min before infection, viral replication was reduced, with respect to control infected cells, as shown by all the four assays performed (Figure 7A–D). However, both SeDA4 and SeMC actually retained their capacity to promote viral replication even after oxidative stress super-induction by H_2_O_2,_ in the two assays in which they were tested (Figure 7C,D). Conversely, when the effects of the selenium-based compounds where assayed in DN-IκB U937 cells infected by HSV-1, i.e. in cells in which HSV-1-induced ROS production was inhibited by ablation of NF-κB activation, no changes in ROS production (Figure 8A,B), as well as in viral replication, as detected by fluorescence microscopy analysis of gD positive cells (Figure 8C), were observed. The latter results demonstrated that the selenium-amino acids, at the utilized concentrations, did not exert by themselves any promoting or inhibiting effect on HSV-1 replication in monocytic cells. 

### 3.6. Pre-Treatment with Selenium-Based Antioxidants Inhibits NF-κB Activation in Monocytic Cells Infected by HSV-1

**Figure 9:**
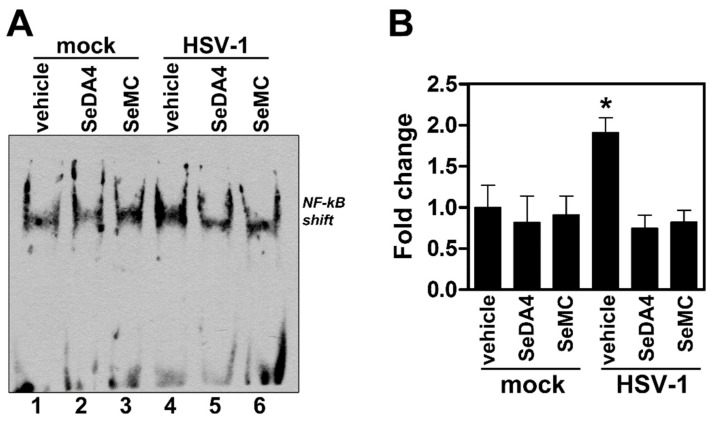
Effects of selenium-based antioxidants on HSV-1-induced NF-kB activation. Nuclear extracts from U937 cells treated with SeDA4 (20 µM), SeMC (20 µM) or vehicle alone for 48 h and then exposed or mock-exposed to HSV-1 MOI 50 for further 1 h, were prepared for NF-kB DNA activity by EMSA. (**A**) EMSA gel from one representative experiment. The position of NF-kB DNA shift is indicated. (**B**) Quantitative evaluation of EMSA by densitometry image analysis. Results are expressed as fold change in shift band intensities from all sample versus corresponding mock-infected, vehicle treated samples. Each bar represents the mean value + S.D. of three independent experiments. * P < 0.001 vs mock-infected, vehicle treated samples.

HSV-1 has been previously demonstrated to trigger NF-κΒ activation by a signaling pathway involving oxidative stress in macrophages [32]. We then asked whether such a phenomenon could occur even in our experimental model of HSV-1 infection, characterized by the NF-κB-dependency of ROS production. To this purpose, we assayed levels of NF-κB activation by EMSA in U937 cells pretreated or not with SeDA4 or SeMC and successively infected by HSV-1. Figure 9 shows that the selenium-based antioxidants actually exerted an inhibitory effect towards HSV-1-induced NF-kB activation, indicating a strict relationship between the phenomenon of inhibition of ROS production and that of inhibition of NF-kB activation induced by SeDA4 and SeMC in HSV-1-infected cells.

### 3.7. HSV-1-Induced ROS Production and Related Effects Are Monocytic Cell Type-Dependent

**Figure 10:**
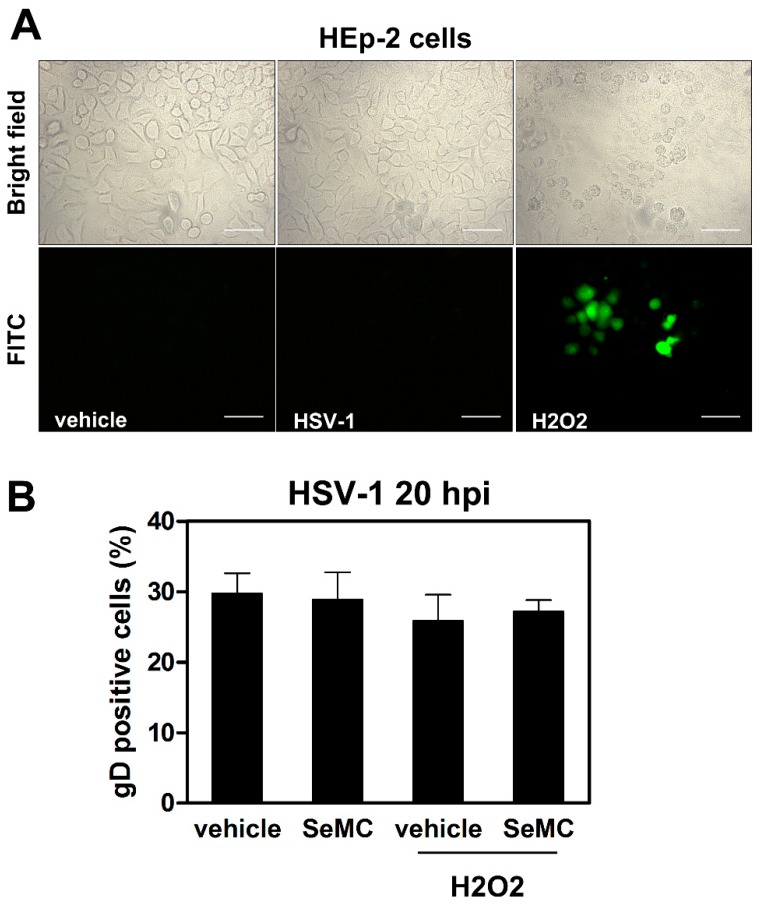
ROS modulation in HEp-2 cells infected by HSV-1. (**A**) Fluorescence microscopic analysis of cells preloaded with DCFH-DA before exposure to HSV-1 (MOI = 10 PFU/cell) for 2 h or treatment with 5 µM of H_2_O_2_ for 30 min. Objective: 40×. Scale bar = 50 μm. (**B**) Evaluation of gD-HSV-1 expressing cells by immunofluorescence microscopy analysis of cells cultured with vehicle or SeMC (20 µM) for 48 h and then treated or not with H_2_O_2_ (5 µM) for 30 min before infection with HSV-1 (MOI = 0.5 PFU/cell). Cells were collected at 20 h post infection. Analysis by IFA is expressed as % positive cells (mean + S.D.) calculated from 10 different fields per conditions of two independent experiments. No statistically significant differences were found between different conditions.

Having demonstrated a noticeable, complex interplay among NF-κB activation, ROS production and HSV-1 replication in monocytic cells, we were interested in establishing whether the observed phenomena could be restricted to cells of the specific, monocytic cell type utilized in our experiments, which are not fully permissive to HSV-1. ROS generation and effects of selenium-based compounds on viral replication were then assayed in the fully permissive HEp-2 epithelial cell line infected by HSV-1. Figure 10 shows that neither ROS production in response to infection (Figure 10A) nor any change in viral replication after SeMC treatment, as detected by gD expression (Figure 10B), could be detected in HEp-2 cells infected by HSV-1. Moreover, unresponsiveness of HEp-2 cells to produce ROS following HSV-1 infection was not caused by a lack in the oxidative machinery, as shown by a well detectable oxidative response to H_2_O_2_ super-induction (Figure 10A). Finally, differently from what occurred in monocytic cells, no effects of SeMC on viral replication, even after H_2_O_2_ super-induction, were observed in HEp-2 cells (Figure 10B). All together, these results account for a cell type-dependency of the interplay between NF-κB activation and ROS production in HSV-1 infected cells.

## 4. Discussion

Existence of a complex, not univocal, interplay between NF-κB activation and oxidative stress, and awareness that this interplay could impact on the innate response, have been known for years [33,34,35,36]. Moreover, evidence for such an interplay in response to HSV-1 infection of macrophages has been already given [32]. In the present study, however, we addressed for the first time the issue of the cross-talk between NF-κΒ activation and oxidative stress in the framework of mechanisms that underlie the restriction of HSV-1 infection in monocytic cells. Actually, it is rather obvious that restriction of HSV infection in monocytes/macrophages plays a pivotal role in preventing the catastrophic scenario of massive destruction by HSV once in contact with this component of the innate immune defence. Nevertheless, although HSV infection of mononuclear monocytic/phagocytic cells has been described by years, few data are available on complex mechanisms underlying the full or partial restrictiveness of HSV infection in these cells [10,18,37,38]. In fact, results we obtained provide some simple, but solid, novel findings that could contribute in understanding as to why the progression of HSV-1 infection is so different in fully permissive epithelial cells and not in fully permissive monocytic cells.

A novel finding from our study is that the immediate oxidative stress response to HSV-1 in monocytic cells is NF-κB-dependent. Even if, based on the results obtained by other authors in different, not virus-related, experimental systems, this is not fully surprising, it is the first time that this is demonstrated for ROS-induction by HSV-1. However, we must consider that our observation is strictly related to the monocytic cell type and cannot be generalized to all types of HSV infections. Interestingly, in fact, we detected a similar, prompt oxidative burst in response to HSV-1 infection in the two monocytic cell lines examined, but not in epithelial fully permissive HEp-2 cells in which, notoriously, NF-κB activation is equally induced by the virus [5]. Nevertheless, considering the pivotal role of NF-κB activation on restriction of HSV-infection of monocytic cells and that the early activation of this transcription factor occurs immediately after the first contact of the virus with the HVEM receptor in cells expressing this receptor, as we demonstrated [31], our novel finding suggests by itself a possible link between the oxidative stress response and the NF-κB-dependent modulation of HSV-1 infection. Interestingly, moreover, we observed that the state of hypo-responsiveness to oxidative stress in DN-IκB cells is not restricted to HSV-1-induced triggering but also concerns changes in the intracellular redox status caused by exposure to non-toxic doses of H_2_O_2_. However, further deepening of this aspect and possible mechanisms involved, is out of the purpose of this study.

A second, well depicted, novel finding we want to highlight is the demonstration that pre-treatment of monocytic cells with compounds having the capability to inhibit NF-κB-dependent ROS production, and no direct effect on virus replication as shown by experiments on DN-IκB cells, unequivocally enhanced viral replication in HSV-1 infected monocytic cells. Sporadic data indicating that an oxidative stress response could be associated with an antiviral, innate immunity in HSV-1 infected cells have been previously published [26]. However, these studies were focused on the innate response signalling cascade and not explicitly on their effects on virus replication. Actually, studies focused on the effects of antioxidants on HSV viral replication led to the conclusion that inhibition of the oxidative stress response in HSV infected cells suppressed HSV DNA synthesis, presumably in a NF-κB-dependent manner [39,40]. These results, apparently, strongly contrast with those reported by us in this study. However, we must take into consideration that our study and those reported above were carried out in completely different cellular systems, such as permissive epithelial cells and not fully permissive monocytic cells, as in our study.

Relevant to this apparent discrepancy between our results and those reported by others, we must note that previous studies by us and other authors, indicate that NF-κB activation has an enigmatic dual face in HSV-1 infection. In fact, on one hand, NF-κB activation represents a subtle form of viral immune evasion that redirects the cellular response to infection to a mechanism for favouring virus replication in fully permissive cells [11]. Conversely, on the other hand, activation of NF-κB can act as a cellular antiviral tool to antagonize infection, as shown by existence of specific anti- NF-κB HSV-1 proteins [41] and by our finding that ablation of NF-κB activation promotes virus replication in monocytic cells [19]. Relevant to the latter is that HSV-1 replication, under normal conditions, progresses in a self-limiting manner in monocytic cells, as well as in other immune cell types. This consideration prompted us to hypothesize that the dual role of NF-κB activation towards HSV-1 replication could be related to the specific cell type in which the virus, once entered, attempts to complete its replication cycle [19]. Unfortunately, the mechanisms capable to explain the differential outcome of NF-κB activation in fully permissive or not fully permissive cell types were not identified.

Considering that the results reported by us in the present study define a strict interplay between NF-κB activation and ROS production in monocytic cells infected by HSV-1, we can assume that the different outcome of antioxidants on HSV-1 replication in epithelial cells and monocytic cells reflects differences observed on the role of NF-κB activation in cells that are fully permissive to HSV-1. Further investigation is necessary to confirm this inference and elucidate involved mechanisms. However, relevant to the above assumption could be our, apparently puzzling, finding that not only ROS production in response to HSV-1 in monocytic cells is NF-κB dependent, but also that NF-κB activation induced by HSV-1 is favoured by oxidative stress, as shown in Figure 9. Moreover, our experiments on CHX-treated cells revealed that the oxidative burst triggered by HSV-1 in monocytic cells is not dependent on de novo protein synthesis. This suggests the hypothesis that in monocytic cells immediately after the first contact with HSV-1 virions, presumably in response to HSV-1 glycoprotein D/HVEM interaction, an interactive loop involving both NF-κB activation and ROS production is established and this loop could contribute to determining a status of antiviral innate response that can limit virus replication in these cells. At the same time, the antioxidant machinery of the cells should be alerted and activated in order to contain the effects of this loop and to avoid the detrimental consequences for the cells of an excessive and prolonged oxidative burst. Our results show that the transient detection of ROS in HSV-1 infected monocytic cells could account for this self-limiting mechanism. The balance between antiviral factors and proviral immune evasion factors could differ at the single cell level and this could explain why, in a minority of the monocytic cells, HSV-1 is able in any case to complete its replication cycle. Moreover, we must consider that the transcription factor NF-κB exerts a fundamental role also in controlling cell death machinery during infection [19]. As a consequence, restriction of HSV-1 infection in monocytic cells could be the result of multifactorial events in which the NF-κB/oxidative stress cross-talk could play a central role. In addition, we cannot help but notice that our data indicate that a pharmacological inhibition of the oxidative stress could promote HSV-1 replication in restricted monocytic cells, adding new questions on the use of antioxidant drugs during HSV-1 infection.

In conclusion, results reported in the present study show that a strict link exists between NF-κB activation and the oxidative burst in response to HSV-1 infection of monocytic cells. Our results, although still leaving open questions, depict a novel scenario and offer the basis for further investigation that could contribute to understanding the mechanisms involved in restriction of HSV-1 infection in mononuclear immune cells.

## Figures and Tables

**Table 1:** Primers utilized for the Nox family genes.

Nox Family Component	Gene Name	GeneBank Number		Sequence (5’→3’)
Nox2/gp91phox	CYBB	NM_000397	F	CTTCCTCAGGGGTTCCAGTG
			R	GTGAATCGCAGAGTGAAGTGC
Nox4	NOX4	NM_001143837	F	TCCAGCCTCCAGAACTTACTTC
			R	ACCTCTTCTTTGCGTCCACT
p47phox	NCF1	NM_000265	F	GAGAGCGGTTGGTGGTTCTG
			R	CACCTGCATAGTTGGGCTCA

## References

[1] Wertheim J.O., Smith M.D., Smith D.M., Scheffler K., Kosakovsky Pond S.L. (2014). Evolutionary origins of human herpes simplex viruses 1 and 2. Mol. Biol. Evol..

[2] Roizman B., Whitley R.J. (2013). An inquiry into the molecular basis of HSV latency and reactivation. Annu. Rev. Microbiol..

[3] Gnann J.W., Whitley R.J. (2017). Herpes Simplex Encephalitis: An Update. Curr. Infect Dis. Rep..

[4] Mancini M., Vidal S.M. (2018). Insights into the pathogenesis of herpes simplex encephalitis from mouse models. Mamm Genome.

[5] Amici C., Belardo G., Rossi A., Santoro M.G. (2001). Activation of I kappa b kinase by herpes simplex virus type 1. A novel target for anti-herpetic therapy. J. Boil. Chem..

[6] Amici C., Rossi A., Costanzo A., Ciafre S., Marinari B., Balsamo M., Levrero M., Santoro M.G. (2006). Herpes simplex virus disrupts NF-kappaB regulation by blocking its recruitment on the IkappaBalpha promoter and directing the factor on viral genes. J. Boil. Chem..

[7] Gianni T., Leoni V., Campadelli-Fiume G. (2013). Type I interferon and NF-kappaB activation elicited by herpes simplex virus gH/gL via alphavbeta3 integrin in epithelial and neuronal cell lines. J. Virol..

[8] Goodkin M.L., Ting A.T., Blaho J.A. (2003). NF-kappaB is required for apoptosis prevention during herpes simplex virus type 1 infection. J. Virol..

[9] Gregory D., Hargett D., Holmes D., Money E., Bachenheimer S.L. (2004). Efficient replication by herpes simplex virus type 1 involves activation of the IkappaB kinase-IkappaB-p65 pathway. J. Virol..

[10] Mastino A., Sciortino M.T., Medici M.A., Perri D., Ammendolia M.G., Grelli S., Amici C., Pernice A., Guglielmino S. (1997). Herpes simplex virus 2 causes apoptotic infection in monocytoid cells. Cell Death Differ..

[11] Patel A., Hanson J., McLean T.I., Olgiate J., Hilton M., Miller W.E., Bachenheimer S.L. (1998). Herpes simplex type 1 induction of persistent NF-kappa B nuclear translocation increases the efficiency of virus replication. Virology.

[12] Sciortino M.T., Medici M.A., Marino-Merlo F., Zaccaria D., Giuffre M., Venuti A., Grelli S., Mastino A. (2007). Signaling pathway used by HSV-1 to induce NF-kappaB activation: Possible role of herpes virus entry receptor D. Ann. N Y Acad. Sci..

[13] Sciortino M.T., Medici M.A., Marino-Merlo F., Zaccaria D., Giuffre-Cuculletto M., Venuti A., Grelli S., Bramanti P., Mastino A. (2008). Involvement of gD/HVEM interaction in NF-kB-dependent inhibition of apoptosis by HSV-1 gD. Biochem. Pharmacol..

[14] Yedowitz J.C., Blaho J.A. (2005). Herpes simplex virus 2 modulates apoptosis and stimulates NF-kappaB nuclear translocation during infection in human epithelial HEp-2 cells. Virology.

[15] Liu X., Fitzgerald K., Kurt-Jones E., Finberg R., Knipe D.M. (2008). Herpesvirus tegument protein activates NF-kappaB signaling through the TRAF6 adaptor protein. Proc. Natl. Acad. Sci. USA.

[16] Pelton B.K., Imrie R.C., Denman A.M. (1977). Susceptibility of human lymphocyte populations to infection by herpes simplex virus. Immunology.

[17] Daniels C.A., Kleinerman E.S., Snyderman R. (1978). Abortive and productive infections of human mononuclear phagocytes by type I herpes simplex virus. Am. J. Pathol..

[18] Linnavuori K., Hovi T. (1981). Herpes simplex virus infection in human monocyte cultures: Dose-dependent inhibition of monocyte differentiation resulting in abortive infection. J. Gen. Virol..

[19] Marino-Merlo F., Papaianni E., Medici M.A., Macchi B., Grelli S., Mosca C., Borner C., Mastino A. (2016). HSV-1-induced activation of NF-kappaB protects U937 monocytic cells against both virus replication and apoptosis. Cell Death Dis..

[20] Yang Y., Bazhin A.V., Werner J., Karakhanova S. (2013). Reactive oxygen species in the immune system. Int. Rev. Immunol..

[21] Matsuzawa A., Saegusa K., Noguchi T., Sadamitsu C., Nishitoh H., Nagai S., Koyasu S., Matsumoto K., Takeda K., Ichijo H. (2005). ROS-dependent activation of the TRAF6-ASK1-p38 pathway is selectively required for TLR4-mediated innate immunity. Nat. Immunol..

[22] Tal M.C., Sasai M., Lee H.K., Yordy B., Shadel G.S., Iwasaki A. (2009). Absence of autophagy results in reactive oxygen species-dependent amplification of RLR signaling. Proc. Natl. Acad. Sci. USA.

[23] Gonzalez-Dosal R., Horan K.A., Rahbek S.H., Ichijo H., Chen Z.J., Mieyal J.J., Hartmann R., Paludan S.R. (2011). HSV infection induces production of ROS, which potentiate signaling from pattern recognition receptors: Role for S-glutathionylation of TRAF3 and 6. PLoS Pathog..

[24] Gonzalez-Dosal R., Horan K.A., Paludan S.R. (2012). Mitochondria-derived reactive oxygen species negatively regulates immune innate signaling pathways triggered by a DNA virus, but not by an RNA virus. Biochem. Biophys. Res. Commun..

[25] Kavouras J.H., Prandovszky E., Valyi-Nagy K., Kovacs S.K., Tiwari V., Kovacs M., Shukla D., Valyi-Nagy T. (2007). Herpes simplex virus type 1 infection induces oxidative stress and the release of bioactive lipid peroxidation by-products in mouse P19N neural cell cultures. J. Neurovirol..

[26] Chen X., Qiao H., Liu T., Yang Z., Xu L., Xu Y., Ge H.M., Tan R.X., Li E. (2012). Inhibition of herpes simplex virus infection by oligomeric stilbenoids through ROS generation. Antivir. Res..

[27] Nucci A., Marino-Merlo F., De Nisco M., Pedatella S., Rossi F., Jacob C., Caputo R., Mastino A. (2014). Se-(2-aminoalkyl)selenocysteines as biochemical redox agents. A tool to contrast cell injury induced by aflatoxin B1 in HepG2 cells. Amino Acids.

[28] Medici M.A., Sciortino M.T., Perri D., Amici C., Avitabile E., Ciotti M., Balestrieri E., De Smaele E., Franzoso G., Mastino A. (2003). Protection by herpes simplex virus glycoprotein D against Fas-mediated apoptosis: Role of nuclear factor kappaB. J. Boil. Chem..

[29] Marino-Merlo F., Papaianni E., Maugeri T.L., Zammuto V., Spano A., Nicolaus B., Poli A., Di Donato P., Mosca C., Mastino A. (2017). Anti-herpes simplex virus 1 and immunomodulatory activities of a poly-gamma- glutamic acid from Bacillus horneckiae strain APA of shallow vent origin. Appl. Microbiol. Biotechnol..

[30] Matteucci C., Minutolo A., Balestrieri E., Marino-Merlo F., Bramanti P., Garaci E., Macchi B., Mastino A. (2010). Inhibition of NF-kappaB activation sensitizes U937 cells to 3’-azido-3’-deoxythymidine induced apoptosis. Cell Death Dis..

[31] Sciortino M.T., Medici M.A., Marino-Merlo F., Zaccaria D., Giuffre-Cuculletto M., Venuti A., Grelli S., Mastino A. (2008). Involvement of HVEM receptor in activation of nuclear factor kappaB by herpes simplex virus 1 glycoprotein D. Cell Microbiol..

[32] Mogensen T.H., Melchjorsen J., Hollsberg P., Paludan S.R. (2003). Activation of NF-kappa B in virus-infected macrophages is dependent on mitochondrial oxidative stress and intracellular calcium: Downstream involvement of the kinases TGF-beta-activated kinase 1, mitogen-activated kinase/extracellular signal-regulated kinase kinase 1, and I kappa B kinase. J. Immunol..

[33] Morgan M.J., Liu Z.G. (2011). Crosstalk of reactive oxygen species and NF-kappaB signaling. Cell Res..

[34] Nakajima S., Kitamura M. (2013). Bidirectional regulation of NF-kappaB by reactive oxygen species: A role of unfolded protein response. Free. Radic. Biol. Med..

[35] Sies H., Berndt C., Jones D.P. (2017). Oxidative Stress. Annu. Rev. Biochem..

[36] Buelna-Chontal M., Zazueta C. (2013). Redox activation of Nrf2 & NF-kappaB: A double end sword?. Cell Signal.

[37] Kim E.T., White T.E., Brandariz-Nunez A., Diaz-Griffero F., Weitzman M.D. (2013). SAMHD1 restricts herpes simplex virus 1 in macrophages by limiting DNA replication. J. Virol..

[38] Sciortino M.T., Perri D., Medici M.A., Grelli S., Serafino A., Borner C., Mastino A. (2006). Role of Bcl-2 expression for productive herpes simplex virus 2 replication. Virology.

[39] Chen D., Su A., Fu Y., Wang X., Lv X., Xu W., Xu S., Wang H., Wu Z. (2015). Harmine blocks herpes simplex virus infection through downregulating cellular NF-kappaB and MAPK pathways induced by oxidative stress. Antivir. Res..

[40] Faith S.A., Sweet T.J., Bailey E., Booth T., Docherty J.J. (2006). Resveratrol suppresses nuclear factor-kappaB in herpes simplex virus infected cells. Antivir. Res..

[41] van Lint A.L., Murawski M.R., Goodbody R.E., Severa M., Fitzgerald K.A., Finberg R.W., Knipe D.M., Kurt-Jones E.A. (2010). Herpes simplex virus immediate-early ICP0 protein inhibits Toll-like receptor 2-dependent inflammatory responses and NF-kappaB signaling. J. Virol..

